# Cohort Profile Update: The Irish Longitudinal Study on Ageing (TILDA)—Waves 5 and 6

**DOI:** 10.1093/ije/dyaf158

**Published:** 2025-09-28

**Authors:** Siobhan Scarlett, Ann Monaghan, Sinead McLoughlin, Ann Hever, Cathal McCrory, Mark Ward, Christine A McGarrigle, Rose Anne Kenny

**Affiliations:** The Irish Longitudinal Study on Ageing (TILDA), Medical Gerontology, School of Medicine, Trinity College, Dublin, Ireland; The Irish Longitudinal Study on Ageing (TILDA), Medical Gerontology, School of Medicine, Trinity College, Dublin, Ireland; The Irish Longitudinal Study on Ageing (TILDA), Medical Gerontology, School of Medicine, Trinity College, Dublin, Ireland; The Irish Longitudinal Study on Ageing (TILDA), Medical Gerontology, School of Medicine, Trinity College, Dublin, Ireland; The Irish Longitudinal Study on Ageing (TILDA), Medical Gerontology, School of Medicine, Trinity College, Dublin, Ireland; The Irish Longitudinal Study on Ageing (TILDA), Medical Gerontology, School of Medicine, Trinity College, Dublin, Ireland; The Irish Longitudinal Study on Ageing (TILDA), Medical Gerontology, School of Medicine, Trinity College, Dublin, Ireland; The Irish Longitudinal Study on Ageing (TILDA), Medical Gerontology, School of Medicine, Trinity College, Dublin, Ireland; Mercer’s Institute for Successful Ageing, St James’s Hospital, Dublin, Ireland

**Keywords:** longitudinal data, ageing, epigenetics, demographics, population, Ireland, data linkage

Key Features

- The Irish Longitudinal Study on Ageing (TILDA)—a nationally representative cohort of 8175 community-dwelling adults aged ≥50 years—was established in 2006 to capture the social, health, and economic circumstances of the ageing population in Ireland.
- Six waves of data collection have been completed, after a pilot phase, with the most recent wave taking place in 2021–2023 during the COVID-19 pandemic, offering a unique opportunity to analyse the impact of this global health crisis.
- Because of the COVID-19 pandemic, the sixth wave of data collection required significant reconfiguration of fieldwork modes. For the first time, interviews were conducted via telephone, self-completion questionnaires (SCQs) were distributed by post, and saliva and faecal sample kits were posted for self-collection and postal return.
- Additional new sub-studies were undertaken during the sixth wave, such as a COVID-19 SCQ, saliva and faecal sample collections, and new modalities of cognitive assessments for the TILDA Healthy Cognitive Ageing Project.
- Information on data access for research and research collaboration can be found at https://tilda.tcd.ie/data/accessing-data/ or by emailing the senior data manager, Dr Siobhan Scarlett (siobhan.scarlett@tcd.ie).

## The original cohort

The Irish Longitudinal Study on Ageing (TILDA) is a nationally representative longitudinal study of community-dwelling adults residing in Ireland [1]. The study has been described in detail previously [1, 2]. The first wave of data collection took place between 2009 and 2011. The sampling frame was drawn from the Irish Geodirectory—a comprehensive listing of all residential addresses in Ireland. Addresses were randomly sampled by using the Random Sample (RANSAM) method [3], meaning that each address had an equal probability of selection. Individuals at these addresses aged ≥50 years and capable of providing informed consent were invited to take part in the study. Spouses of any age were also invited.

There were three modes of assessment during the Wave 1–5 fieldwork periods. At each wave, a face-to-face computer-assisted personal interview (CAPI) was administered by a trained field interviewer and participants were invited to fill in a self-completion questionnaire (SCQ) [2, 4]. In alternate waves, a comprehensive health assessment (HA) was administered by a health practitioner in the TILDA health assessment centre or a shorter assessment was carried out in the participant’s own home [1, 2, 4]. A fourth mode of assessment was introduced during Wave 6 fieldwork—a computer-assisted telephone interview (CATI).

Participants who experience cognitive or physical impairment that may compromise their ability to complete a self-report interview are offered a proxy interview instead where consent has previously been obtained. Where participants have passed away, an end-of-life (EOL) interview is completed with a close family member or friend of the participant.

## What is the reason for the new focus?

The study has now completed six waves of data collection, spanning >15 years since the first interview was completed. A Cohort Profile Update was published in 2018 covering the period up to the completion of Wave 4 data collection [1]. The purpose of this update is to describe developments and innovations during Wave 5 (2018) and Wave 6 (2020–2023). This review encompasses new sub-studies; adaptations to fieldwork during COVID-19 lockdown periods; and the ongoing enhancement of the TILDA study through data linkage and biomarker processing. A replenishment sample was also recruited as part of Wave 6 for new participants aged 45–64 years.

### Fieldwork reconfiguration

Preparation for fieldwork generally commences 12 months in advance of the wave. Fieldwork for Wave 6 commenced in January 2020 but was suspended in March 2020 due to the COVID-19 outbreak. Extensive periods of movement restrictions were in place during 2020 and 2021 that limited face-to-face interaction. TILDA therefore opted to reconfigure the mode of assessment for Wave 6 from CAPI to CATI in 2021 and delay the HA and cohort replenishment to 2022, as initial face-to-face contact was considered optimal for potential new participants.

## What will be the new areas of research?

TILDA is now a well-established longitudinal study and described in Kenny *et al.* [2] and Donoghue *et al.* [1]. Each wave of data collection introduces new content to ensure that topical information is captured. New introductions at Wave 5 included information on pneumococcal vaccinations, e-cigarette usage, public-transport usage, membership of sports clubs, technology usage, discrimination, pet ownership, and sleep chronotypes. Wave 6 (2020–2023) captured information on COVID-19, including participant circumstances, experiences due to public health restrictions, and personal infection, vaccination uptake, and attitudes. Wave 6 additionally acted as a test bed for methodological adaptations for pandemic preparedness. The sixth wave of data collection also marked the third longitudinal wave of HAs, providing an opportunity for rich analyses of ageing trajectories.

More specifically, areas of research to be focused on include:

- epigenetics and omics profiling, including metabolomics, genomics, transcriptomics, and proteomics [5, 6];
- impact of the COVID-19 pandemic by using a COVID-19 SCQ and saliva samples measuring a suite of SARS-CoV-2 antibodies [7];
- social connectedness [8];
- lifelong environmental exposures [9, 10];
- cognitive decline and accelerated brain ageing [11–15];
- projection of care needs and the role of the caregiver [16, 17];
- unmet healthcare need (through comparison of objective health measurements coupled with self-reported diagnoses), disease clusters, and multimorbidity [18];
- falls risk and prevention.

Additional detail is provided in the Supplementary Material of enhanced areas of research and data processing (e.g. biomarker and molecular data) (Supplementary Methods S1).

## Who is in the cohort?

**Figure 1.**
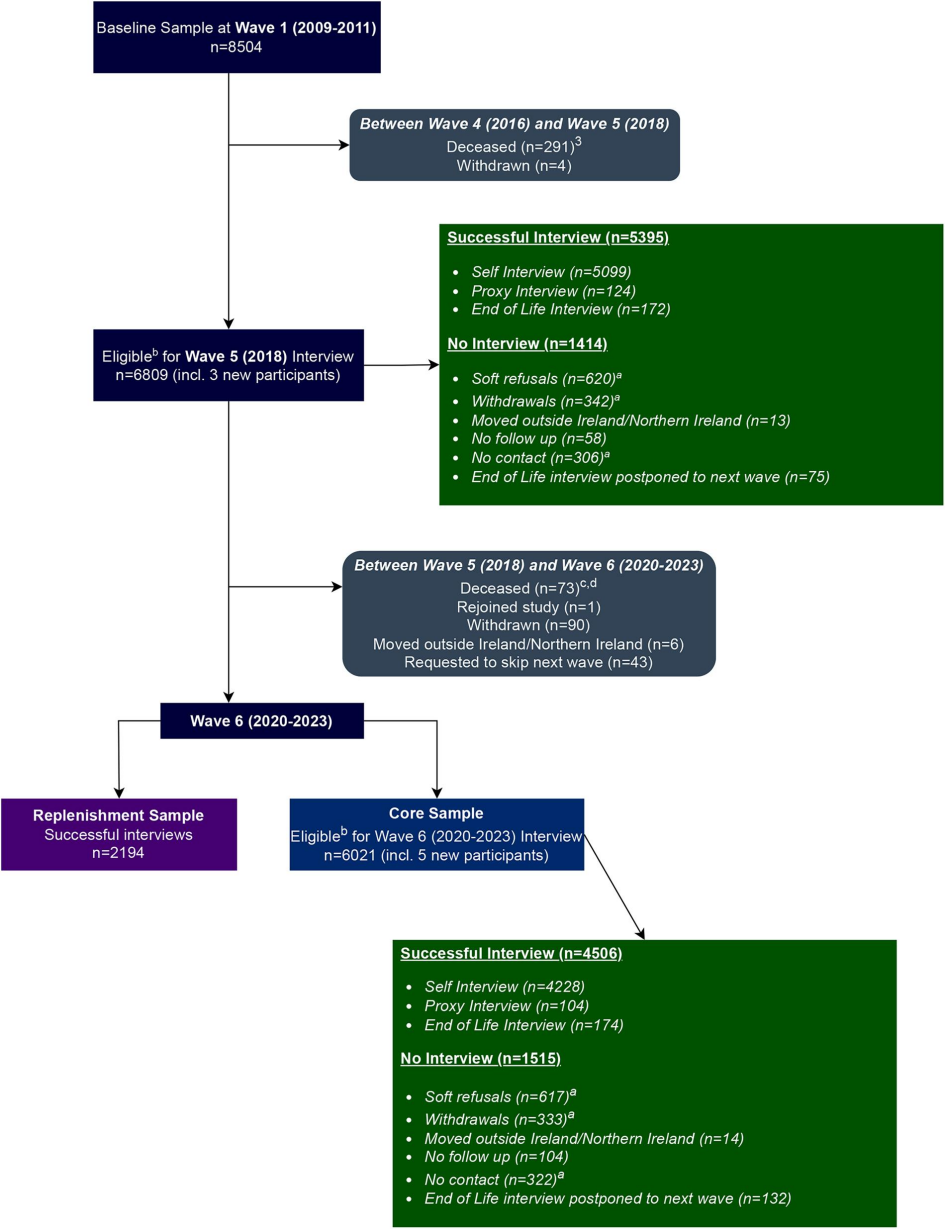
Attrition flowchart following the completion of Wave 4 to the end of Wave 6 showing sample eligibility and completed interviews. Detailed breakdowns of attrition between waves, such as withdrawals and descriptions of attrition during each wave are provided. ^a^Soft refusals: participants who opted to skip the wave of data collection but remained in TILDA for subsequent waves of data collection; Withdrawn: participants who requested to leave TILDA entirely and refused all future data collection; No contact: participants who were uncontactable at the time of data-collection fieldwork after five or more attempts. ^b^Fieldwork sample eligibility: participants who have withdrawn, have moved outside Ireland/Northern Ireland, have been marked as no follow-up (e.g. proxy participants who did not give permission for a proxy interview), and those who request to skip a wave will not be included in the eligible fieldwork sample at sample drawdown for each wave. ^c^Deceased participants are included in the eligible fieldwork sample until an EOL interview has been completed or the participant has been marked as no follow-up where an EOL interview has been refused or no individual was available to complete the interview on behalf of the participant. ^d^A group of deceased participants were excluded from the eligible fieldwork sample for Wave 6 as they were identified as deceased after the fieldwork for the EOL interviews, which were separated from the self-interviews for this wave, had already been completed.

Wave 1 included 8504 (8175 aged 50+) participants. Sample attrition from Waves 1 to 4 is covered in detail in the previous Cohort Profile Update [1]. Figure 1 presents updated numbers covering the baseline sample and attrition after completion of Wave 4 to the end of Wave 6. In Wave 5, 5223 participants completed a self-report/proxy interview and 172 EOL interviews were completed. In Wave 6, 4332 of the core sample completed a self-report/proxy interview, 174 EOL interviews were completed, and 2194 of the newly recruited replenishment sample completed an interview.

### Replenishment sample

A total of 2194 participants from 1708 households were recruited between May 2022 and October 2023 in Wave 6 as the sample replenishment.

To recruit the replenishment sample, the RANSAM sampling procedure was followed [2, 3]. Households identified in the original sampling frame who did not have eligible participants or were held in reserve and not visited in the first wave of data collection were revisited. The eligibility criteria for replenishment closely matched those of the core sample but with lower baseline age (to aide interview uptake, as recruitment proved difficult in the early stages of fieldwork) and more restricted range. Eligibility criteria were:

- community-dwelling;
- aged 45–64 years (and spouse/partner of any age);
- cognitively and physically capable of providing informed consent.

Descriptive statistics of the Wave 1 sample (as described in Donoghue *et al.* [1]), Wave 6 sample, replenishment sample, and the combined sample are provided in Table 1. The largest shifts in prevalence between the baseline sample and Wave 6 were, by design, higher proportions of 45- 65-year-olds. Notably, more participants in Wave 6 had tertiary and secondary education compared with those in Wave 1, partly driven by the introduction of free secondary education in 1966 (Table 1).

**Table 1.** Participant characteristics of the baseline sample of TILDA, the core and replenishment sample at Wave 6, and the combined sample at Wave 6. Demographic breakdowns of age, sex, education, marital status, and location are provided. Descriptive statistics of the Wave 1 sample are adapted from Donoghue *et al.* [1]. Appropriate permission for reproduction has been obtained.

	Completed self-report interview at Wave 1	Completed self-report interview at Wave 6 (core sample)	Completed self-report interview at Wave 6 (replenishment sample)	Completed self-report interview at Wave 6 (combined sample)
	*n* = 8504	*n* = 4228	*n* = 2194	*n* = 6422
Age (years) [*n* (%)]				
<50	330 (3.9)	11 (0.3)	469 (21.4)	480 (7.5)
50–64	4668 (54.9)	985 (23.3)	1630 (74.4)	2615 (40.7)
65–74	2164 (25.4)	1886 (44.6)	91 (4.2)	1977 (30.8)
75+	1342 (15.8)	1345 (31.8)	2 (0.1)	1347 (21.0)
Age (years), mean ± SD	63.1 ± 10.2	71.1 ± 8.0	55.0 ± 6.0	65.6 ± 10.6
Sex [*n* (%)]				
Male	3780 (44.5)	1816 (43.0)	862 (39.3)	2678 (41.7)
Female	4724 (55.5)	2412 (57.1)	1332 (60.7)	3744 (58.3)
Education [*n* (%)]				
Primary	2521 (29.7)	715 (16.9)	72 (3.3)	787 (12.3)
Secondary	3431 (40.4)	1691 (40.0)	578 (26.4)	2269 (35.3)
Tertiary	2548 (30.0)	1822 (43.1)	1542 (70.4)	3364 (52.4)
Marital status [*n* (%)]				
Married	5966 (70.2)	2922 (69.1)	1657 (75.5)	4579 (71.3)
Never married	791 (9.3)	329 (7.8)	271 (12.4)	600 (9.3)
Separated/divorced	552 (6.5)	308 (7.3)	210 (9.6)	518 (8.1)
Widowed	1195 (14.0)	669 (15.8)	56 (2.6)	725 (11.3)
Location [*n* (%)]				
Dublin city or county	2012 (23.7)	1044 (24.7)	572 (26.1)	1616 (25.2)
Other town/city	2390 (28.1)	1142 (27.0)	916 (41.8)	2058 (32.1)
Rural	4102 (48.2)	2042 (48.3)	706 (32.2)	2748 (42.8)

Response rates for the Wave 5 and 6 data-collection periods are presented in Table 2.

**Table 2.** Response rates for Waves 5 and Wave 6 of TILDA including sub-studies run concurrently with the wave.

	Wave 5	Wave 6
	January 2018–December 2018	September 2020–December 2023
	*N* (response rate %)^a^	*N* (response rate %)^a^
**CAPI/CATI** ^a^		
EOL	172 (58)	174 (51)
Self-report	5099 (81)	4228 (76)
Proxy	124 (57)	104 (68)
SCQ^b^	4410 (86)	3501 (83)
Faecal microbiome pilot^c^	n/a	391 (79)
Replenishment SCQ^a^	n/a	1583 (73)
**TILDA Healthy Cognitive Ageing Project** ^d^	n/a	1344 (73)
**HA** ^e^	n/a	1918 (77)
Centre	n/a	1414 (57)
Home	n/a	504 (20)
Blood samples^f^	n/a	2706 (62.5)
TruCulture^g^	n/a	100 (100)
Accelerometry^g^	n/a	1387 (72)
Faecal microbiome^g^	n/a	1499 (78)
Oral HA^g^	n/a	1090 (77)

a Response rate calculated based on eligible participants at each respective wave and interview type (self-reported interview, proxy interview, EOL interview).

b SCQs were only provided to participants who completed a self-report interview at each respective wave.

c Pilot faecal sample collection was offered to a limited number of participants who completed a Wave 6 CATI.

d TILDA Healthy Cognitive Ageing Project (HCAP) (randomly selected subsample of participants aged ≥65 years drawn from the Wave 6 CATI).

e HAs were only offered to participants who completed a Wave 6 CATI self-report interview and had not been invited to take part in the TILDA-HCAP assessment.

f Blood samples were taken from participants who completed either a HA or TILDA-HCAP assessment [15].

g HA sub-studies were offered to different groups of participants: faecal sample collection was offered to all HA participants; accelerometry was offered to all HA participants where a device was available at the end of their assessment; TruCulture^®^ was offered to a limited number of centre participants aged >65 years who met thresholds during their GAITRite and grip strength assessments; oral HAs were offered to all centre participants where a dentist was available.

### Wave 5

The Wave 5 data collection, from January to December 2018, included a CAPI and SCQ for all eligible participants. The CAPI self-report interview and SCQ response rates were 81% and 86%, respectively [19].

### Wave 6

**Figure 2.**
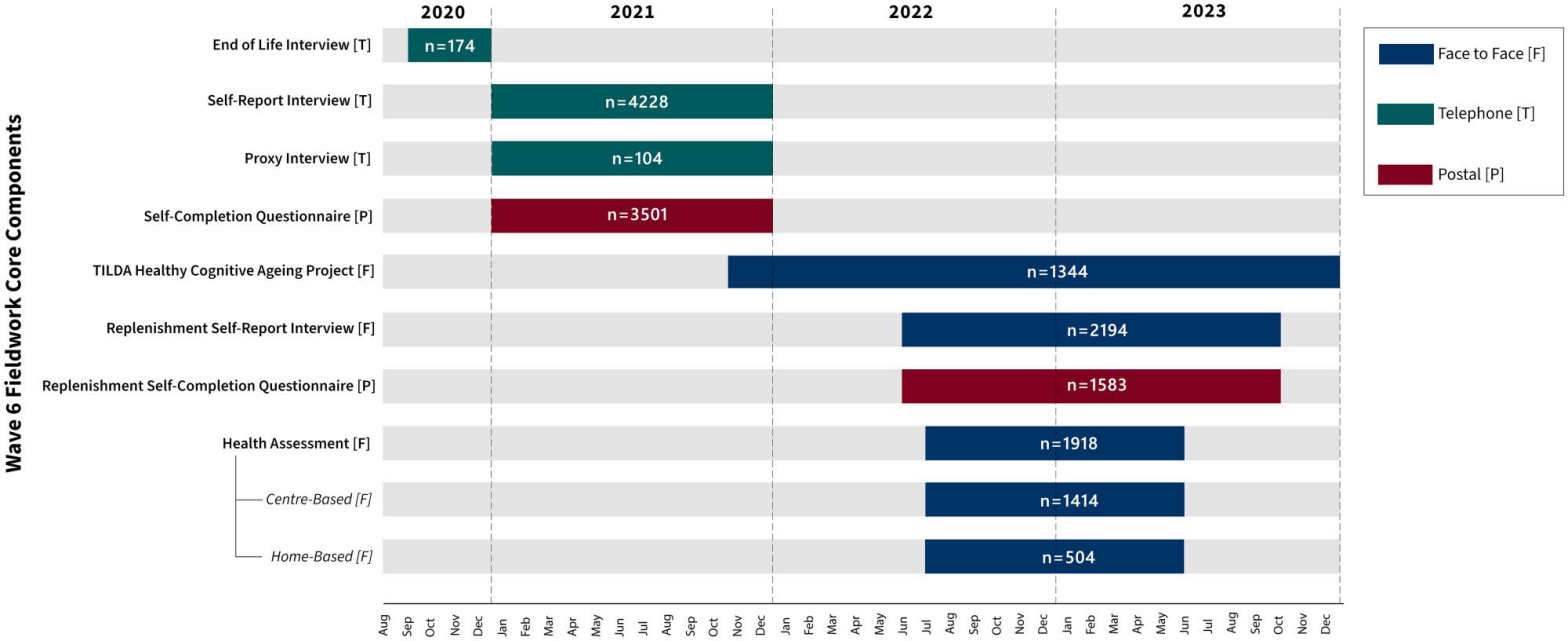
Wave 6 core components timeline including mode of assessment and number of valid responses. Each component of the fieldwork is shown relative to the time span during which the fieldwork took place, with a visual representation of the mode of assessment.

The Wave 6 data collection took place from September 2020 to December 2023. Components were administered as face-to-face (CAPI or health practitioner assessment), telephone (CATI), or postal. A timeline of the core fieldwork components, most of the assessments, and the number of participants is shown in Fig. 2.

Modifications to the self-report interview were required where components could not be administered over the telephone. Physical measures of grip strength and timed up-and-go (TUG) were omitted. The Mini-Mental State Examination (MMSE) (PAR, Inc^®^) [20] has components that require visual assessment by the interviewer, which is not feasible via telephone. To determine cognitive ability, all participants completed the Abbreviated Mental Test, which could be administered over the telephone and assesses cognitive function by using simple verbal tasks, such as memory recall and orientation questions, without requiring physical interaction or visual cues [21].

The self-report interview response rate was lower than those in previous waves, at 76%, as was the SCQ, at 83%. Seventy-six per cent of the sample completed an HA, which was slightly lower compared with previous waves. Twenty per cent of this group opted to complete a home-based rather than centre-based assessment HA.

#### Wave 6 sub-study data collection

Sub-studies ran concurrently with the Wave 6 data collection by using either the CATI or HA samples as a base for eligible participants (Supplementary Methods S2). These included a pilot study of faecal microbiome collection on a subsample of CATI participants (*n *= 391), subsequently offered to all HA participants. Additional blood samples were also drawn from a subsample of HA participants (*n *= 100) as part of the TruCulture^®^ sub-study for innate immune analysis.

## What has been measured?

An overview of the topics collected in each core component is provided in Table 3. Topics remain largely consistent between waves with exclusions where change over time would be minimal and immediate longitudinal follow-up is not necessary (e.g. sleep chronotype). Novel topics included those of relevance to the pandemic in Wave 6. All questions and topics for inclusion are decided before each wave by consensus of researchers, external stakeholders, healthcare professionals, policy makers, funders, and participant members of Participant and Public Involvement (PPI) group. For example, inclusion of pet ownership at Wave 5 was a consequence of PPI recommendation [22]. Inclusion of questions and topics depends on time constraints, forthcoming policy initiatives, scientific innovation, and societal need.

**Table 3.** Data/tests collected in the TILDA interview, SCQ, and HAs from Waves 1–6. Overall domains and individual components within each domain are provided.

CAPI/CATI	Wave 5	Wave 6 core	Wave 6 replenishment
**Demographics**			
Household composition^1–4^	✓	✓	✓
Marital status^1–4^	✓	✓	✓
Education^1–4^	✓	✓	✓
Childhood circumstances^1–4^	✓	✓	✓
Siblings information^1–4^	✓	✓	✓
Migration history^1–4^	✓	✓	✓^lf^
**Social circumstances**			
Financial and non-financial assistance given and received^1–4^	✓	✓	✓
Social connectedness^1–4^	✓	✓	✓
Helpers^1–4^	✓	✓	✓
Religion^1–4^	✓	✓	
Volunteering and caring^1–4^	✓	✓	✓
Crime^3,4^	✓		
Driving and travel^1^		✓	✓
**Literacy**			
Numeracy/financial literacy^3,4^	✓		✓^lf^
**Retirement and expectations**			
Planning for retirement^1–4^	✓	✓	✓^lf^
Expectations^1–4^	✓	✓	
Advance care planning^4^		✓	✓
**Economic circumstances**			
Employment situation^1–4^	✓	✓	
Job history^1–4^	✓	✓	✓^lf^
Pensions^1–4^	✓	✓	✓^lf^
Sources of income^1–4^	✓	✓	✓
Household consumption^3–4^	✓	✓	✓
Assets^1–4^	✓	✓	✓^lf^
House ownership^1–4^	✓	✓	
**Physical health**			
Self-rated health^1–4^	✓	✓	✓
Sensory function (smell/taste/sight/hearing)^1–4^	✓	✓	✓
Functional limitations^1–4^	✓	✓	✓
(Instrumental) activities of daily living^1–4^	✓	✓	✓
Cardiovascular conditions^1–4^	✓	✓	✓
Chronic conditions^1–4^	✓	✓	✓
Falls/fractures^1–4^	✓	✓	✓
Fear of falling^1–4^	✓	✓	✓^lf^
Pain^1–4^	✓	✓	✓
Vaccination^1–4^	✓	✓	✓
Healthcare screening^1–4^	✓	✓	✓
Healthcare utilization^1–4^	✓	✓	✓
Medications^1–4^	✓	✓	✓
Physical function: TUG^2,4^	✓		✓^lf^
Physical function: grip strength^2,4^	✓		
**COVID-19**		✓	✓
Behavioural health			
Smoking^1–4^	✓	✓	✓
Physical activity^1–4^	✓	✓	✓
Sleep^1–4^	✓	✓	✓
Alcohol use^2–4^^,^^a^	✓	✓	
**Cognitive health**			
Self-rated memory^1–4^	✓	✓	✓
MMSE^2–4^	✓	✓	✓
Verbal fluency^1–4^	✓	✓	✓
Immediate and delayed recall^1–4^	✓	✓	✓
Prospective memory^1–4^	✓		✓
**Mental health**			
Self-reported mental health^1–4^	✓	✓	✓
Life satisfaction^1–4^	✓	✓	✓
Depression^1–4^	✓	✓	✓
Anxiety^2–4^	✓	✓	✓

SCQ	Wave 5	Wave 6 core	Wave 6 replenishment
Social activities^1–4^	✓	✓	✓
Clubs/organizations^4^	✓	✓	✓
QOL^1–4^	✓	✓	✓
Loneliness^1–4^	✓	✓	✓
Stress^1–4^	✓	✓	✓
Relationship quality^1–4^	✓	✓	✓
Alcohol use^1–4^	✓	✓	✓
Ageing perceptions^1,3^	✓	✓	✓
Worry (Penn State)^1–4^	✓	✓	✓
Technology and internet use	✓	✓	✓
Discrimination	✓	✓	✓
Heating^2–4^	✓	✓	✓
Social standing^4^	✓		✓
Pet ownership	✓		✓
Sleep (Munich Chronotype Questionnaire)	✓		✓
Creative activity		✓	✓
Anxiety^1^		✓	✓
Falls efficacy^2,3^		✓	✓
Stress^3^		✓	✓
Life satisfaction		✓	✓
Fruit and vegetable consumption^3,4^		✓	✓
COVID-19		✓	✓
International Physical Activity Questionnaire (IPAQ)—Environmental		✓	✓
Purpose in life^4^		✓	✓
Gambling		✓	
Stressful life events^1,2^			✓
Sexual activity^2,4^			✓
Neighbourhood disorder^2,4^			✓
Childhood health conditions^4^			✓

EOL interview	Wave 5	Wave 6 core	Wave 6 replenishment
**Demographics**			
Circumstances of death^2–4^	✓	✓	–
Residence before death^2–4^	✓	✓	–
**Physical, mental, and cognitive health**			
(Instrumental) activities of daily living^2–4^	✓	✓	–
Helpers^2–4^	✓	✓	–
Chronic illness^2–4^	✓	✓	–
Falls/fracture^2–4^	✓	✓	–
Pain^2–4^	✓	✓	–
Cognitive function^2–4^	✓	✓	–
Mood^2–4^	✓	✓	–
**Behavioural health**			
Alcohol use^2–4^	✓	✓	–
Smoking^2–4^	✓	✓	–
Weight^2–4^			
Healthcare utilization^2–4^	✓	✓	–
Assets and life insurance^2–4^	✓	✓	–
Advance/after death planning^3–4^	✓	✓	–
COVID-19		✓	–

Wave 6 HA	Wave 6 Core	
	Centre	Home
**Neuropsychological tests**		
MMSE^1,3^	✓	✓
Montreal Cognitive Assessment (MOCA)^1,3^	✓	✓
Sustained Attention to Response Task (SART)^1,3^	✓	✓
Choice reaction time^1,3^	✓	✓
Colour trails^1,3^	✓	✓
Centre for Epidemiological Studies Depression scale (CES-D) short form^1,3^	✓	✓
State anxiety^3^	✓	✓
**Cardiovascular tests**		
Blood pressure (OMRON)^1,3^	✓	✓
Pulse wave velocity^1,3^	✓	
Heart rate variability^1,3^	✓	
Phasic blood pressure^1,3^	✓	
Near infrared spectroscopy (NIRS)^3^	✓	
**Physical function**		
TUG^1,3^	✓	✓
Repeated chair stands^3^	✓	✓
**GAITRite**		
Normal pace^1,3^	✓	
Dual task (cognitive)^1,3^	✓	
Maximum pace^3^	✓	
**Ultrasound**		
Heel bone ultrasound^1,3^	✓	
**Anthropometric/other**		
Grip strength^1,3^	✓	✓
Height and weight^1,3^	✓	
Waist circumference^1,3^	✓	✓
Bloods^1,3^	✓	✓
Faecal sample	✓	✓
Oral health^3^	✓	
Accelerometry^3^	✓	✓

[1–4] indicate the previous waves in which the measure has been included.

lf A long-form version of the replenishment interview was administered to 183 participants before the interview was shortened to improve response rates and encourage wider participation in the sampling frame. This measure was only included in the long-form replenishment interview.

a Alcohol use in CAPI/CATI questionnaire is only asked of proxy respondents.

b Fruit and vegetable consumption was measured as part of a food frequency questionnaire in Waves 3 and 4.

### Data linkage

Data linkage with administrative and health datasets continued throughout 2019–2023 with the Primary Care Reimbursement Scheme database [23], General Registry Office death records [8], General Practitioner (GP) electronic medical records, and sources of environmental and spatial data for spatial analysis [9, 10]. These projects are described in Supplementary Methods S3.

## What has it found? Key findings and publications

As of December 2024, the TILDA study data had been used in >600 publications (https://tilda.tcd.ie/publications/publications-search/). TILDA has also been cited in key discussions on health policy and planning in the Irish Parliament. A search of the Parliament (Oireachtas) debates for the term ‘The Irish Longitudinal Study on Ageing’ yielded 143 mentions [24]. Below are samples of new findings since the previous Cohort Profile Update, including the output on social epidemiology, predictors of mortality, health outcomes, biomarkers, accelerometry, and MRI data.

A strong association between social disconnection and a wish to die was identified, as was a bidirectional association between loneliness and depression, suggesting interventions enabling prosocial settings and alleviating loneliness may be protective against suicidal ideation [25, 26]. McGarrigle *et al.* (2023) highlighted that, in contrast to the focus on negative outcomes in caregiving, analyses of TILDA carers showed resilient trajectories around life satisfaction, noting the importance of support networks [17].

Projections of care needs showed that older people living with a serious disease requiring palliative care will increase by >80% between 2016 and 2046, highlighting an urgent need for improved resources, funding, and service provisions [16]. Strong disease clusters were confirmed, highlighting important issues for healthcare service and policies as the complexities for care needs increase with multimorbidity [18].

Combining the mortality information with TILDA’s novel DNA methylation data found that the GrimAge epigenetic clock outperformed other established clocks in predicting age-related decline and all-cause mortality [5]. Subsequent research on the epigenetic data highlighted the importance of early-life deprivation and metabolic syndrome as important predictors of biological ageing [27, 28].

Delayed recovery of systolic blood pressure upon standing predicted accelerated progression of age-related macular degeneration and accelerated brain ageing [14]. For the first time in a large representative sample, normative reference values for frontal lobe oxygenation when resting and during an active stand were reported for clinical use by using near infrared spectroscopy [11]. Impaired frontal lobe perfusion was also a predictor of clinically relevant depression and generalized anxiety disorder [29, 30].

Low folate predicted accelerated cognitive decline over 8 years, with implications for public policies on folic acid fortification [12]. Higher levels of hair cortisol and cortisone (measures of oxidative stress) were associated with poorer cognitive performance [13], while hair cortisol and the cortisol-to-cortisone ratio were associated with cerebral oxygenation [31].

## What are the main strengths and weaknesses?

TILDA is now a well-established longitudinal study, featuring six waves of data collection and HA data at three distinct time points. The study has become a valuable resource for informing policies on all aspects of societal ageing at the population level. A rapid reconfiguration of methodology was successfully applied to ensure that fieldwork could continue during COVID-19 restrictions, with lessons learned for future pandemic preparedness. In recognition of the quality of the work and the international impact of TILDA, it has been designated as the WHO Collaborating Centre for Longitudinal Studies on Ageing and the Life Course, playing a crucial role in advancing knowledge and data on ageing and life-course research, with a focus on supporting ageing studies in low- and middle-income countries (https://tilda.tcd.ie/who-collaborating-centre/terms/).

One of the key strengths of the TILDA study is the breadth of objective HA data. Comparing self-report versus objectively measured outcomes allows a thorough assessment of unmet need, thus assisting healthcare providers and systems to identify high-risk groups, target interventions, and address gaps in treatment. The expansion of biomarkers, including epigenetics and metabolomics at Waves 1 and 3, affords more precise health and lifespan profiling and risk stratification [32], also furthering understanding of the relationship between early-life adversity and the pace of biological ageing [27].

Limitations are also present. Additional processing of the biomarkers, such as for epigenetics, is expensive, and preparation of the samples and appropriate paperwork are resource-intensive. To date, these measures have only been available for a subset of participants at each wave so far.

While TILDA has made progress with data linkage to external databases, the lack of an individual health identifier in Ireland limits the ability to conclusively link certain datasets. Therefore, while consent for GP linkage is high (∼90%), the linkage process, coordination with GP practices, and data preparation are cumbersome and slow.

As part of the replenishment of participants aged 45–64 years, TILDA endeavoured to recruit a population-representative cohort within this age demographic. The replenishment cohort has an overrepresentation of females (60% vs 50%) and is underrepresented in the non-White Irish population (11% versus 19.96% compared with Census 2022 figures in this age cohort). Consequently, this may limit the generalizability of findings. Survey weights have been established to account for this disparity in future iterations of the datasets.

## Can I get hold of the data? Where can I find out more?

Researchers can apply to access pseudonymized TILDA data through the Irish Social Science Data Archive (https://www.ucd.ie/issda/) at University College Dublin. Datasets are deposited 18–24 months following the completion of fieldwork. Pseudonymization techniques, such as top and bottom coding, aggregation, and small-cell-size removal, have been applied to these datasets to ensure that participants are not directly identifiable. Approved researchers can request to access more detailed versions of the dataset, including additional variables, either through an in-person hotdesk facility (for researchers based in Ireland), or remotely via a Trusted Research Environment, TILDA VISTA (https://tilda.tcd.ie/tilda-vista/). TILDA also run regular in-person and online workshops to assist researchers in navigating the dataset. Information regarding accessing TILDA data and TILDA workshops can be found on the TILDA website (https://tilda.tcd.ie/data/). Queries about data access can be sent to the TILDA senior data manager, Dr Siobhan Scarlett (siobhan.scarlett@tcd.ie).

## Ethics approval

TILDA Wave 5 data collection was approved by the Trinity College Dublin Faculty of Health Sciences Research Ethics Committee on 15 May 2017 (Ref: 170304) and for Wave 6 on 31 May 2019 (Ref: 190407).

## Supplementary Material

dyaf158_Supplementary_Data

## Data Availability

Details of how to access the TILDA data are outlined in the section ‘Can I get hold of the data?’ above. Enquiries about data access can also be sent to either tilda@tcd.ie or Dr Siobhan Scarlett, at siobhan.scarlett@tcd.ie.
