# Supplementary material for: Cohort Profile Update: The Irish Longitudinal Study on Ageing (TILDA)—Waves 5 and 6
Source: Int J Epidemiol. 2025 Sep 28;54(5):dyaf158. doi: 10.1093/ije/dyaf158 (PMC12476908; doi:10.1093/ije/dyaf158)
Supplement: dyaf158_Supplementary_Data [file dyaf158_supplementary_data.docx]

**Supplementary Material**

**Glossary of Terms**

| CAPI | Computer Assisted Personal Interview |
| --- | --- |
| CATI | Computer Assisted Telephone Interview |
| EOL | End of Life |
| GP | General Practitioner |
| HA | Health Assessment |
| HCAP | Healthy Cognitive Ageing Project |
| MMSE | Mini-Mental State Examination |
| PPI | Patient & Participant Involvement |
| SCQ | Self-Completion Questionnaire |
| TILDA | The Irish Longitudinal Study on Ageing |
| TUG | Timed-Up and Go |

**Supplementary Methods S1: Expanded detail of new areas of research**

Wave 6 included a series of sub-studies to enhance the data collection. A suite of viral antibodies including five SARS-CoV-2 antigens and four seasonal Coronavirus Spike proteins were measured from salivary samples; faecal samples were collected for future gut microbiome analyses; markers of innate immunity analysed from additional blood draws; and an SCQ on behaviours and attitudes and well-being during the pandemic^1-3^.

TILDA maintains onward processing of data collected from previous waves. This includes detailed spatial analysis for information on environmental exposomes and proximity to amenities, such as healthcare facilities; processing of magnetic resonance imaging (MRI) data collected in Wave 3^4^ including global and regional volume and thickness; Scheltens’ scores; connectivity matrices; Diffusion tensor magnetic resonance imaging (DTI); white matter hyperintensities; grey/white matter cerebral blood flow; Arterial spin labelling (ASL) MR perfusion^5-10^ and a novel measure of brain age-measures of BrainPAD^11^ . Accelerometry data (*n*=1614) collected in Wave 3^12^ includes measures of sleep duration and disruption, sedentary behaviour and physical activity^13-15^, with ongoing sleep efficiency, latency, awakenings, daytime napping, and sleep stage classification processing. Wave 6 also used accelerometry (*n*=1291) for longitudinal comparisons.

Expansion of biomarker and molecular data includes Metabolomics, providing insight by detailed metabolite profile and altered metabolomic functions associated with age and age-related disease phenotypes. Metabolomic profiling was completed on 1400 of the Wave 1 baseline sample and 500 repeat samples at the Wave 3 using the National Phenome Centre’s (NPC) established platform for liquid chromatography–mass spectrometry (LC-MS)-based global profiling of molecules and lipids. To date, 700 metabolites have been extracted related to age-related features.

Blood draws have been collected for future analyses of genomics, transcriptomics, and proteomics coupled with enhanced numbers for further DNA methylation analyses following the paradigm flow of information from gene (DNA) to transcript (mRNA) to protein.

**Supplementary Methods S2. Description of TILDA Sub-studies**

*COVID-19 Self Completion Questionnaire: Altered lives in a time of crisis: preparing for recovery from the impact of the COVID-19 pandemic on the lives of older adults*

A SCQ was designed to capture information on the circumstances of TILDA participants during the first COVID-19 lockdown between July and November 2020. Specifically, the aims of the study were to:

(1) provide a description of the prevalence of COVID-19 symptomatology and testing among older adults.­­
(2) describe levels of adherence to public health guidelines intended to halt the spread of the virus; (3) examine health-related, caring, and other unmet needs.
(4) measure changes in well-being and examine whether these vary between groups defined by gender and other socio-demographic characteristics, socioeconomic status, coexisting conditions, and existing psychological ill-health.
(5) examine whether older adults experienced ageism or discrimination during the pandemic.
(6) describe how public health information was received and understood.

The COVID-19 Self Completion Questionnaire key findings report and data management plan has been published describing the methodology in detail^16, 17^.

*SARS-CoV-2 specific AntiBodieS in The Irish Longitudinal Study on Ageing (TILDA-SABS)*

The aim of the study was to determine seroprevalence rates of SARS-CoV-2 in older adults in Ireland and explore demographic, clinical and biological factors associated with COVID-19 disease trajectory in the TILDA cohort. A major challenge faced early in the project was that we could not employ the traditional approach of measuring SARS-CoV-2 antibodies in blood samples from participants, due to ongoing pandemic-related restrictions preventing us from meeting participants face-to-face to collect a blood sample. Therefore, we collaborated with a research team from the Institut Pasteur, Paris, who had developed a novel method to measure a suite of SARS-CoV-2 antibodies in saliva samples. The sponge swab, used similarly to a toothbrush, collects saliva by firmly rubbing along the gumline, extracting both saliva and gingival crevicular fluid (GCF, rich in Immunoglobulin G (IgG)). This non-invasive, user-friendly process involves placing the swab back into its container after collection.

Participants were posted the saliva kit in January 2021, immediately prior to vaccine roll-out, along with a short COVID-19 questionnaire with questions pertaining to whether the participant had been infected with COVID-19, the symptoms they experienced, close contacts and likelihood of receiving a vaccine when available. Participants returned the saliva sample, questionnaire, and consent form using the provided pre-paid envelope. Samples were extracted and the saliva was biobanked and the team used a multiplex ELISA approach to quantify levels of SARS-CoV-2 and related seasonal coronavirus antibodies in the saliva of TILDA participants.

*TILDA Healthy Cognitive Ageing Project (TILDA-HCAP)*

The Harmonised Cognitive Assessment Protocol (HCAP) was developed and validated by the US Health and Retirement Study (HRS) to support data harmonisation. These resources will enhance research opportunities by leveraging cross-country differences in crucial life-course factors that impact cognitive function and Alzheimer’s Disease (AD)/Alzheimer’s Disease and Related Dementias (ADRD) risk, such as education, wealth, retirement policies, diet, and cardiovascular risk factors. TILDA, part of the HRS family, is unique in collecting additional objective measures of cardiovascular, autonomic, neuropsychological data, and sensory measures. This extra information, harmonised with HCAP measures, will improve understanding of risk factors and cognitive biomarkers in future HRS family survey waves participating in HCAP.

TILDA-HCAP assessments took place between December 2021 and January 2024. The study aimed to complete 1,800 assessments with a projected response rate of 80%. Those who were invited for a HCAP assessment would not be invited for the core health assessment. Participants eligible for inclusion were those aged 65 years or older by December 2021 and who had conducted a Wave 6 self-interview. The sample to be invited for assessment included all participants who were living alone, and a random selection of 50% of participants who were married.

Assessments were conducted by health practitioners in the participant’s home.

The Detailed TILDA-HCAP methodology, and initial results can be found in the cohort profile, published elsewhere^18^.

*Faecal sample collection*

The purpose of faecal sample collection was to determine gut microbiome diversity in the TILDA population. It has been widely reported that gut microbiome diversity declines with age. Conversely, disease causing pathogenic bacterial species increase with age. These maladaptations of the gut microbiome with ageing, referred to as dysbiosis, have recently been highlighted as one of the twelve ‘Hallmarks of Ageing’^19^. Variation in the gut microbiome is minimally driven by genetics and is largely influenced by the exposome - environmental, behavioural and socio-economic factors.

As with the SABS-TILDA study, in-person data collection was not feasible due to ongoing population-level restrictions on movement and travel. TILDA therefore opted to administer postal-based sample collection kits to participants. Prior to data collection, TILDA undertook a feasibility assessment with the TILDA PPI group. Key outcomes for this assessment were ease of use of sample kits and readability and clarity of instructions. 100% agreed to provide a faecal sample to TILDA in future; 84% found the collection kit easy to use; and 66% found the instructions easy to use. Instructions were updated in accordance with PPI group recommendations.

Faecal samples were self-collected by participants using at-home collection kits (OMNIgene GUT sample collection kit and postal accessories (DNA Genotek, Ottawa, Canada). Participants were instructed to place a pea-sized sample into the collection tube, and to package in accordance with instructions in the information leaflet. Participants were also asked to complete a microbiome SCQ, which included questions on GI conditions and surgeries, infectious GI illness, antibiotic and laxative medications, short food frequency questionnaire, prebiotic and probiotic consumption, and the date and time of sample collection. A free text box was also provided for additional feedback. All participants were provided with appropriate packaging and envelopes and were asked to post samples to TILDA, along with the questionnaires on the same day the sample was taken. Returned faecal samples and questionnaires were received by the TILDA Biobank Laboratory, recoded with a study ID number and the samples stored at -80oC. All samples were further processed into two 1ml aliquots in 2ml cryovial tubes within 12 months and biobanked at -80oC. The questionnaires were returned securely to the TILDA Data Team and were coded, quality checked and the data entered to create an electronic dataset.

Collection of TILDA participant faecal samples was repeated in the Wave 6 health assessment with all participants now invited to take part, including those who had been invited previously as part of the CATI sub-study. The sample kit, instructions, and return procedures remained the same.

*TruCulture*

This study aimed to assess how innate immune responses, namely anti-viral type I interferon (IFN-I) and inflammatory responses, are activated and regulated in older adults. TILDA, as a member of the Milieu Interior (MI) Consortium (https://www.milieuinterieur.fr/en/), used similar methodology to the MI study to assess these responses which will therefore allow for comparison to their younger, healthier cohort to provide a wider lifespan approach to this investigation. Blood for a select cohort of participants in the Wave 6 TILDA health assessment will be stimulated for 22h with innate immune agonists using the TruCulture immunomonitoring system. After stimulation, cell pellets will be stored at -80ºC for downstream transcriptomic analysis and supernatants will be stored at -80ºC for downstream proteomics analysis.

The criteria for inclusion in the TruCulture study was male and female participants aged between 70-79 years old, with either a slow Timed up and Go (TUG) score (>10.5 seconds), or a fast TUG score (≤9 seconds) in a prior wave, and all participants aged 80+ years. Participants also had to have a recorded TUG during their Wave 6 health assessment. Participants were identified in advance of their health assessment as meeting the criteria for the TruCulture study. Each participant was asked to provide an additional 12ml blood sample (3 tubes x 4ml) if they consented to blood sample collection as part of their assessment.

**Supplementary Methods S3. Description of TILDA Linkage Projects**

*TILDA-PCRS*

The Primary Care Reimbursement Service (HSE) is a reimbursement scheme managed by the Health Service Executive. A range of medications are provided through the medical card and other drugs schemes. The PCRS database hosts extensive information on pharmaceutical use of all users of the medical card scheme. TILDA has requested consent for linkage to the PCRS database from participants with a medical card at all waves.

To date, TILDA has completed linkage for Wave 1 to Wave 5, covering PCRS usage from 2008-2022. PCRS linkage provides extensive data on claim dates, drug type, dosage, providing objective medication use information for participants using the medical card scheme.

*TILDA-GRO*

The General Register Office (GRO) is a repository of civil records including death records. The death record provides the official date of death, direct cause of death, antecedent causes of death, and any other significant conditions. TILDA has completed two linkages with the GRO records covering deaths in the cohort up to November 2021. Only participants who were active in the study at the time of their passing are included in the linkage requests.

*TILDA-GP*

TILDA requested consent for matching with General Practitioner (GP) records in Wave 5 of the Core sample and also the Replenishment sample. The linkage is a collaboration between TILDA and the Royal College of Surgeons in Ireland (RCSI). The process for linkage is strictly controlled given the sensitivity of the information being transferred. The data returned includes extensive objective medical record information pertaining to consultations, investigations, diagnoses, prescriptions, vaccinations and referrals.

*TILDA-GIS*

TILDA has generated extensive spatial data using Geographical Information System software to enhance the TILDA datasets with information around proximity to healthcare services, amenities, transport options, in addition to environmental exposures such as radon, noise pollution and air pollutants such as PM_2.5_. Geocoded location data from each wave of data collection is used to generate this information for analysis of current exposures and accessibility.

TILDA has also collected historical address information from participants as part of the Self-Completion Questionnaire in Wave 3, allowing for generation of spatial data to map historical exposures where historical maps exist. Information such as water sources and exposure to fluoridation can be generated, allowing for rich analysis of impact in later life of early life exposures.

*Gateway Exposome collaboration*

In addition to the GIS information generated to date, an ongoing collaboration with The Gateway to Global Aging Data (g2aging.org) has provided detailed exposome data for linkage with TILDA data. The Gateway to Global Aging Data is a data and information hub funded by the US National Institute of Aging (NIA), whose primary aim is to facilitate cross-study analyses on ageing from its International Network of Studies (HRS-INS). The Gateway team has provided TILDA with harmonised, country-specific maps of environmental exposures that they have derived from satellite data. Maps are processed using geographical information system (GIS) software and mean values of a wide array of exposures for each geolocation are extracted (Table S1).

A policy database has also been established and is maintained by the Gateway to Global Aging team, allowing researchers to use policy variation cross time and location to identify causal impacts of factors such as education, pensions, retirement, care policies etc^20^.

Table S1. Harmonised exposome data provided by the Gateway to Global Aging

| Exposure | Years available |
| --- | --- |
| Particulate matter 2.5 (PM_2.5_) | 2000-2019 |
| Ozone | 1990, 1995, 2000-2021 |
| Light at night | 1995, 2000-2021 |
| Blue space | 2001-2021 |
| Green space | 2001-2012 |
| Nitrogen oxide (NO_2_) | 2005-2017 |
| Precipitation and temperature (forthcoming) | 1990-2022 |

**Supplementary References**

1. Ward M, Clarke N, Wang M, et al. Study protocol for TILDA COVID-19 survey. Altered lives in a time of crisis: preparing for recovery from the impact of the COVID-19 pandemic on the lives of older adults [version 1; peer review: 1 approved, 1 approved with reservations]. *HRB Open Research* 2021; **4**.

2. Hennelly N, Lalor G, Gibney S, Kenny R, Ward M. A cross-sectional study of the relationship between delayed healthcare utilisation and chronic conditions among older adults during the COVID-19 pandemic in Ireland [version 1; peer review: 1 approved, 1 approved with reservations]. *HRB Open Research* 2021; **4**.

3. Ward M, Briggs R, McGarrigle CA, De Looze C, O’Halloran AM, Kenny RA. The bi-directional association between loneliness and depression among older adults from before to during the COVID-19 pandemic. *International Journal of Geriatric Psychiatry* 2023; **38**: e5856.

4. Donoghue OA, McGarrigle CA, Foley M, Fagan A, Meaney J, Kenny RA. Cohort Profile Update: The Irish Longitudinal Study on Ageing (TILDA). *International Journal of Epidemiology* 2018; **47**: 1398-l.

5. Boyle R, Jollans L, Rueda-Delgado LM, et al. Brain-predicted age difference score is related to specific cognitive functions: a multi-site replication analysis. *Brain Imaging and Behavior* 2021; **15**: 327-45.

6. De Looze C, Feeney JC, Scarlett S, et al. Sleep duration, sleep problems, and perceived stress are associated with hippocampal subfield volumes in later life: findings from The Irish Longitudinal Study on Ageing. *Sleep* 2021; **45**.

7. Hirst RJ, Whelan R, Boyle R, et al. Gray matter volume in the right angular gyrus is associated with differential patterns of multisensory integration with aging. *Neurobiol Aging* 2021; **100**: 83-90.

8. Carey D, Nolan H, Kenny RA, Meaney J. Cortical covariance networks in ageing: Cross-sectional data from the Irish Longitudinal Study on Ageing (TILDA). *Neuropsychologia* 2019; **122**: 51-61.

9. Simon C, Bolton DAE, Meaney JF, et al. White matter fibre density in the brain's inhibitory control network is associated with falling in low activity older adults. *European Journal of Neuroscience* 2024; **59**: 3184-202.

10. Leidhin CN, McMorrow J, Carey D, et al. Age-related normative changes in cerebral perfusion: Data from The Irish Longitudinal Study on Ageing (TILDA). *NeuroImage* 2021; **229**: 117741.

11. M AS, O'Connor JD, Boyle R, et al. Slower speed of blood pressure recovery after standing is associated with accelerated brain aging: Evidence from The Irish Longitudinal Study on Ageing (TILDA). *Cereb Circ Cogn Behav* 2024; **6**: 100212.

12. Scarlett S, Nolan H, Kenny RA, O'Connell MD. Objective Sleep Duration in Older Adults: Results From The Irish Longitudinal Study on Ageing. *J Am Geriatr Soc* 2020; **68**: 120-8.

13. Maasakkers CM, Thijssen DH, Knight SP, et al. Hemodynamic and structural brain measures in high and low sedentary older adults. *Journal of Cerebral Blood Flow & Metabolism* 2021; **41**: 2607-16.

14. Maasakkers CM, Claassen J, Scarlett S, et al. Is there a bidirectional association between sedentary behaviour and cognitive decline in older adults? Findings from the Irish Longitudinal Study on Ageing. *Prev Med Rep* 2021; **23**: 101423.

15. Scarlett S, Nolan HN, Kenny RA, O'Connell MDL. Discrepancies in self-reported and actigraphy-based sleep duration are associated with self-reported insomnia symptoms in community-dwelling older adults. *Sleep Health* 2021; **7**: 83-92.

16. Ward M, O'Mahoney P, Kenny RA. *Altered lives in a time of crisis: The impact of the COVID-19 pandemic on the lives of older adults in Ireland. Findings from The Irish Longitudinal Study on Ageing*. Dublin: The Irish Longitudinal Study on Ageing; 2021 2021/01/28/.

17. Ward M, Clarke N, McLoughlin S, Golden D, Kenny R. Data Management Plan for a rapid response sub-study of an existing cohort. The Irish Longitudinal Study on Ageing (TILDA) COVID-19 sub-study [version 1; peer review: awaiting peer review]. *HRB Open Research* 2024; **7**.

18. Feeney J, Monaghan A, McLoughlin S, et al. Cohort Profile Update: The Harmonised Cognitive Assessment Protocol Sub-study of The Irish Longitudinal Study on Ageing (TILDA-HCAP). *International Journal of Epidemiology* 2025; **54**.

19. López-Otín C, Blasco MA, Partridge L, Serrano M, Kroemer G. Hallmarks of aging: An expanding universe. *Cell* 2023; **186**: 243-78.

20. *Gateway to Global Aging - Policy Explorer*. [cited 02/05/2025]; Available from: <https://g2aging.org/gpe/overview2025-05-02>
